# Insights into biodiversity sampling strategies for freshwater microinvertebrate faunas through bioblitz campaigns and DNA barcoding

**DOI:** 10.1186/1472-6785-13-13

**Published:** 2013-04-04

**Authors:** Brandon J Laforest, Amanda K Winegardner, Omar A Zaheer, Nicholas W Jeffery, Elizabeth E Boyle, Sarah J Adamowicz

**Affiliations:** 1 Faculty of Environmental Studies, York University, 4700 Keele Street, Toronto, ON, M3J 1P3, Canada; 2 Department of Biology, McGill University, 1205 Docteur Penfield, Montréal, QC, H2X 2K6, Canada; 3 Department of Integrative Biology, University of Guelph, 50 Stone Rd. E, Guelph, ON, N1G 2W1, Canada; 4 Biodiversity Institute of Ontario, University of Guelph, 50 Stone Rd. E, Guelph, ON, N1G 2W1, Canada

**Keywords:** Ostracoda, Crustacea, Barcoding biotas, Sampling strategy, Bioblitz, Citizen science, Species richness, Zooplankton, Accumulation curves, Subarctic

## Abstract

**Background:**

Biodiversity surveys have long depended on traditional methods of taxonomy to inform sampling protocols and to determine when a representative sample of a given species pool of interest has been obtained. Questions remain as to how to design appropriate sampling efforts to accurately estimate total biodiversity. Here we consider the biodiversity of freshwater ostracods (crustacean class Ostracoda) from the region of Churchill, Manitoba, Canada. Through an analysis of observed species richness and complementarity, accumulation curves, and richness estimators, we conduct an *a posteriori* analysis of five bioblitz-style collection strategies that differed in terms of total duration, number of sites, protocol flexibility to heterogeneous habitats, sorting of specimens for analysis, and primary purpose of collection. We used DNA barcoding to group specimens into molecular operational taxonomic units for comparison.

**Results:**

Forty-eight provisional species were identified through genetic divergences, up from the 30 species previously known and documented in literature from the Churchill region. We found differential sampling efficiency among the five strategies, with liberal sorting of specimens for molecular analysis, protocol flexibility (and particularly a focus on covering diverse microhabitats), and a taxon-specific focus to collection having strong influences on garnering more accurate species richness estimates.

**Conclusions:**

Our findings have implications for the successful design of future biodiversity surveys and citizen-science collection projects, which are becoming increasingly popular and have been shown to produce reliable results for a variety of taxa despite relying on largely untrained collectors. We propose that efficiency of biodiversity surveys can be increased by non-experts deliberately selecting diverse microhabitats; by conducting two rounds of molecular analysis, with the numbers of samples processed during round two informed by the singleton prevalence during round one; and by having sub-teams (even if all non-experts) focus on select taxa. Our study also provides new insights into subarctic diversity of freshwater Ostracoda and contributes to the broader “Barcoding Biotas” campaign at Churchill. Finally, we comment on the associated implications and future research directions for community ecology analyses and biodiversity surveys through DNA barcoding, which we show here to be an efficient technique enabling rapid biodiversity quantification in understudied taxa.

## Background

One of the biggest impediments to conducting large-scale biodiversity surveys lies in the taxonomic identification of target organisms. This is especially true when dealing with microinvertebrates, where defining morphological features are often discernible only through intensive methods such as slide preparation and microscopy. One group of organisms that exemplifies this dilemma is the small-bodied crustacean class Ostracoda. Ostracods are very common in benthic freshwater communities, but also occur in marine, intertidal, or semi-terrestrial environments. They are useful model organisms for studies on various aspects of ecology and evolution [1-5], given the high prevalence of their calcified bivalve shells in the freshwater fossil record as well as their variability in breeding systems [6,7]. In freshwater systems alone, the class Ostracoda has been conservatively estimated to number close to 2,000 described species [8], with 420 freshwater species recorded for North America [9,10]. Taxonomic keys are available to the species level for North America and Europe [10-13], and many surveys describe the regional diversity of the class (e.g. [14-20]). The projected global diversity in all habitat types is estimated to be approximately 13,000 [9].

An infrequently discussed challenge in conducting biodiversity surveys is how to design and implement a suitable sampling strategy. While many studies have compared the efficacy of various field collection methods for capturing accurate estimates of planktonic invertebrate community structure [21-29], there has been little discussion of the idea of sampling strategy as a whole in terms of study objectives, sampling instrumentation, time commitments, adaptation of field methods in response to environmental heterogeneity, and sorting of samples prior to identification both in the field and in the laboratory. Given that the sample size of microinvertebrate community analyses is always much greater than the resources available to identify each individual organism to the appropriate taxonomic level, this sorting of organisms representing the sample community is of utmost importance. Previous studies have demonstrated the presence of cryptic species in microinvertebrates [30-32], and highlight the potential to overlook species with cryptic morphology as well as those with low abundance [33].

Establishing timeframes for microinvertebrate surveys can be linked to many different factors such as limited funding associated with fieldwork, appropriate weather windows for collection, and the availability of trained personnel. These limitations are especially applicable to studies conducted in remote locations, as well as areas of intense seasonality. Conducting fieldwork in these regions should be made as efficient as possible not only to limit associated costs, but to limit human interference on the natural system. Furthermore, while there is discussion in the literature on appropriate standards for comparing sampling strategies for freshwater bodies of various size and habitat diversity [23,29], there is less discussion on the rationale behind intensive sampling. This is a key point as more scientists participate in public research, and more research projects involve an aspect of citizen science.

Citizen science involves collaboration between scientists and volunteers to gather field and observational data [34], and several studies have found that these types of collaborations produce reliable data that would be difficult to gather by any individual research group or scientist [35,36]. For biodiversity studies, citizen science projects often encompass large-scale “bioblitzes” that involve collecting a large number of organisms in a short time period, often as short as a few hours (e.g. http://www.get-to-know.org/bioblitz/). Originally coined in 1996 by Susan Rudy of the U.S. National Park Service, the term bioblitz is now widely employed, with citizen-science bioblitzes recorded in countries such as Canada, New Zealand, Portugal, and Taiwan. The results of bioblitzes are typically not published in the scientific literature, despite their widespread occurrence and potential for inclusion [37]. This may be changing, as demonstrated by the August 2012 issue of *Frontiers in Ecology and the Environment*, a special issue dedicated to the publishing of citizen-science research. For these sampling campaigns to remain an effective and efficient use of citizen-scientist collaborations, sampling strategies and specific objectives that may be served by these efforts should be discussed and evaluated. Here, we quantify and compare the outcomes of different student bioblitzes within the Churchill barcoding biotas campaign to measure collection effort in relation to biodiversity yield.

For animals, DNA barcoding using a region of the mitochondrial gene cytochrome *c* oxidase subunit I (COI) is an increasingly common method both for identifying species and for quantifying provisional species diversity [38-41], and can be used to evaluate and compare sampling strategies for biodiversity surveys. Through separating a sample of organisms into molecular operational taxonomic units (MOTUs), it is possible to calculate provisional species richness without the need for morphology-based identifications. DNA barcoding has been previously employed to build accumulation curves for understudied taxa such as parasitoid wasps (Ichneumonidae, Braconidae, Cynipidae and Diapriidae), with barcode-based accumulation curves indicating higher diversity, but the same shape, than accumulation curves built using morphospecies [42]. While Linnean identifications are useful for community ecology studies, due to the possibility of linking with environmental data, the rapid quantification of biodiversity lends itself nicely to answering questions of species richness, species assemblage patterns, and sampling strategy comparison. As the reference library for the Barcode of Life Data Systems (BOLD) [43] grows, more of these unknown MOTUs will be linked to known species and allow for more sophisticated community ecology questions to be asked.

We employed DNA barcoding to compare five sampling strategies of subarctic freshwater ostracods in Churchill, Manitoba, Canada from 2007–2011, using MOTUs as surrogates for species. The present study does not test the effectiveness of DNA barcoding in recovering species boundaries for this group; rather, we use DNA barcoding as a tool to address our main study objective. We assume here that genetic patterns in the freshwater Ostracoda of Churchill mirror those of other microcrustaceans. For example, studies of the Branchiopoda of Churchill [31], freshwater microcrustaceans of Mexico and Guatemala [44], and marine zooplanktonic ostracods [45] have shown strong separation of described species based upon DNA barcodes.

This study presents an *a posteriori* analysis evaluating the success of five sampling strategies in both capturing and estimating the regional diversity of freshwater ostracods in the Churchill region, as this site was selected for an intensive “barcoding biotas” regional biodiversity survey employing DNA barcoding methods (introduced in [46]). Methodological differences among the sampling strategies prevent analysis into the effect of individual variables on strategy success, but still allow for broad-scale exploration of factors influencing the success of collection events at the scale of bioblitzes. The strategies differed in their primary objectives, duration of time spent sampling, number of sites sampled, and method of sorting of samples prior to analysis and deciding which samples to submit for DNA barcoding analysis. It was predicted that our comparison of these strategies would reveal differences in their effectiveness, yielding useful information for the design of future microinvertebrate surveys, with an emphasis on student or citizen bioblitzes. Previous studies [21-29] have compared sampling methods (e.g. tow nets, D-nets, hand nets) but did not use the same methods to measure or compare sampling effectiveness. By contrast, our study provides evidence that the rationale behind a sampling strategy is as important as the equipment used during bioblitzes (especially those with non-expert volunteers). We suggest that a focus on sampling diverse microhabitats is effective and that having two rounds of specimen selection for DNA barcoding will increase efficiency of molecular resource use for quantifying species diversity.

## Methods

### Description of study site

Five sampling and specimen sorting strategies, identified as time-based (2007), rapid-blitz (2008), liberal re-sort (2007/2008), fixed-protocol field method (2010), and flexible-protocol field method (2011) (Table 1), were used to sample ostracods from freshwater habitats across the landscape near Churchill, Manitoba, Canada (latitude N58°47^′^, longitude W94°11^′^). The habitats sampled included standing and flowing fresh and brackish waters, both permanent and ephemeral. All sampling strategies were conducted during and around July, which is expected to be at the height of freshwater invertebrate activity in Churchill. All specimens were preserved in 95% ethanol within hours of collection.

**Table 1:** Summary of the methods used and numbers of genetic clusters found in each ostracod study

**Project**	**Strategy**	**Dates**	**# of sites**	**Net μm**	**Primary target of collection**	**Number of clusters separated by 2% divergence**	**Number of specimens barcoded**	**Number of clusters captured that only appeared in 1 or 2 sampling strategies**
CHUBL	Time- based effort	July 7–21, 2007	9	100	Ostracoda	18	78	5
SAOST	Rapid Blitz	July 9–17, 2008	14	100	Microcrustacea	18	79	6
COCSA	Liberal re-sort	July 7–21, 2007 & July 9–17, 2008	14	100	Ostracoda (re-sort)	29	124	13
OZFWZ	Fixed- protocol	June 3-Aug 25, 2010	27 (75)	64, 100, 250	Insecta (with mixed invertebrates retained)	17	63	5
OZFWC	Flexible- protocol	July 22-Aug 2, 2011	27 (42)	153	Microcrustacea	29	154	13

Study codes “CHUBL”, “SAOST”, “COCSA”, “OZFWC”, and “OZFWZ” refer to codes for each specimen on BOLD (“Process IDs”), each associated with one strategy. These five study codes can be found in three projects (COCSA, OZFWC and OZFWZ) on the Barcode of Life Database (BOLD Systems: http://www.boldsystems.org). For the column “# of sites”, all values shown without brackets represent the number of sites sampled where ostracods were found. Bracketed values represent the total number of sites for the strategy, including those where ostracods were not found. For the first three strategies, the total number of sites and number of sites with ostracods are the same due to targeted selection of suitable habitats.

### Field sampling strategies and morphological sorting

#### Time-based sampling (2007)

From July 7–21, 2007, we sampled nine sites including four coastal rock pool sites, three tundra ponds, and two flowing freshwater habitats, with a complete focus on ostracod biodiversity. Each site was sampled for up to one hour, or when the specimens collected were thought to accurately represent the ostracod biodiversity of the location based on broad-scale morphological classification, whichever came first. Samples were gathered using a standard home aquarium fish net with a mesh size of 100 μm and a 100 μm D-net employed using a poke-and-collect method. All specimens were live-sorted into gross morphospecies using a standard dissecting microscope (20x magnification) in a field laboratory setting based on colour, size, shape, collection date, and collection site. A total of 21 morphospecies was identified in the field, but this was considered to be a liberal number as morphospecies were numbered for each site and not harmonized regionally across sites. A minimum of one individual per morphospecies per site was included in the genetic analysis, but most cases included at least two individuals of each morphospecies from each site, for a total of 94 specimens. This method was based on the time targets associated with collection, the use of a single methodology during collection, and a reliance on field morphospecies when sorting specimens for analysis.

#### Rapid-blitz sampling (2008)

Over six collecting dates from July 9–17, 2008, we sampled 16 sites including eight tundra or fen ponds, one coastal rock bluff pool, three lakes or reservoirs, and four flowing habitats. A rapid-blitz sampling strategy was employed, with time varying across sites according to habitat size and complexity. At each site, a deliberate, active search was performed to sample the variety of microhabitats present, including both planktonic zones and littoral zones to a depth of approximately 0.5 m. While this strategy did employ different methods across habitats, the variability was unsystematic in that there was no set objective for the study prior to sampling, other than contributing to the general species survey at Churchill, with a focus on microcrustaceans. Therefore, no sampling protocol was developed prior to the collection. An aquarium net of 100 μm mesh size was employed, with the net run through the water column rapidly, over and through vegetation, and over the surface of the substrate. Sampling time was at least 10 minutes per site, with the least time spent at rock bluff pools and more time spent at larger habitats and those with abundant vegetation. Samples were brought back to the field laboratory alive, in water from their own habitat, to be sorted on a light box. From each site, specimens were identified by eye to gross morphospecies (including sorting by size, colour, and shape). The intent was to fill a single plate (95 specimens) for DNA barcoding, with the preliminary results used to inform a second round of sorting of the same samples. Typically, at least two individuals per gross morphospecies per site were selected when available, and sometimes up to five in cases of substantial variability within provisional morphospecies. Selected samples were placed individually into small tubes. This method employed a single method of collection and relied on field morphospecies when sorting specimens for analysis.

#### Liberal re-sort of previous sampling methods (2007/2008)

To test the efficiency of live sorting ostracods into morphospecies in a field laboratory setting immediately following collection, we re-sampled extra specimens from the above two collection efforts, spanning 14 collection sites. We originally did not select these specimens for genetic analysis as it was thought that they represented replicates. We sampled 190 additional individuals from the unused sample pools originally sorted in the field following their collection event (with 93 from the 2007 and 97 from the 2008 samples). Multiple replicates of morphospecies were selected (sample size limited by availability of archived samples), and broader-scale methods of classification of morphospecies were employed as much of the colour had faded from the specimens due to storage. We also avoided one conspicuous species of ostracod (large-bodied, blue morphospecies) which had been overrepresented in previous analyses. This method built upon preliminary evidence of undersampling and liberally included specimens for analysis from the other projects, representing a deviation from previously established methodologies which had placed emphasis on field morphospecies to inform sorting procedure.

#### Fixed-protocol field method (2010)

From early June to late August, 2010, we sampled 75 sites across five freshwater habitat types (30 tundra ponds, 30 coastal rock pools, five shallow lakes, seven creeks, and three points along the Churchill River). Each site was sampled three times throughout the spring/summer season, one sampling event for each site in June, July, and August. The sites were sampled in approximately the same order each month. The sites were sampled for the entire aquatic insect and zooplankton community; thus, ostracods were not the sole or main focus of collection. Of these 75 sites, we found ostracods in only 27, and therefore only these sites were included for further analysis in this study. For each site, we selected a sampling location of 20 metres in length parallel to the water edge/shore and sampled 1.5 metres and 5 metres from the edge along this 20 metre transect. If the habitat was too small to mark out a 20 metre transect, we sampled along a transect that covered the longest length of the habitat. Two collection methods were employed as a sampling strategy for these sites. The first collection method involved moving along the 20 metre transect with a dip net (either 100 μm or 250 μm), disturbing the substrate to acquire benthic organisms, in addition to running the dip net throughout the water column to collect any pelagic species. This walking of the transect was done twice at each site. The second collection method involved tossing a plankton tow net (64 μm) up to 10 m from shore twice from a different point each time along the transect and pulling it back towards the collector. Both of these methods were employed to the best of our abilities at all sites, regardless of habitat type. We immediately separated the ostracods into morphospecies based on site, colour, size, and shape using a light box and a standard dissecting microscope in a field laboratory setting, and preserved them in ethanol. Upon return from the field station, samples were stored at −20°C. Two individuals per morphospecies per sampling event were included in the genetic analyses, for a total of 90 specimens. This field method used a pre-defined protocol at each site without consideration of site-specific appropriateness in an attempt to increase consistency to allow for direct comparisons between sites and over time.

#### Flexible-protocol field method (2011)

Specimens were collected from the planktonic and benthic zones of 20 small freshwater or brackish pools located on rock bluffs along the Hudson Bay coast, and 22 tundra ponds from July 22- August 2, 2011. Sites were sampled using separate planktonic and benthic protocols, with the major focus being on microcrustaceans although other taxa were retained as well. Planktonic samples were collected using a plankton net (153 μm), with a total of three tosses encompassing as many different parts of the water body as possible (varying microhabitats, direction, position along shore). While the 153 μm plankton net was a larger mesh size than the nets used for the other strategies, similar or larger mesh sizes have proven effective in other ostracod surveys [17,18], and this is close to the 100–150 μm range recommended for surveying Ostracoda [10]. For each toss, the net was allowed to sink as close to the bottom as possible (without touching) and then pulled to shore at a slightly upward angle. If the water body was too shallow for a plankton net toss; then collection was conducted with a 100 μm hand net using figure-eight patterns over the accessible parts of the water body. Benthic samples were collected with a 250 μm dip net for water bodies with rocky substrates and with a 153 μm weighted plankton net for softer substrates. For dip net collection, the substrate was kicked up and the net was moved in figure-eight patterns over a 2–3 m transect perpendicular to shore. For plankton net collection, the net sank to the bottom and was pulled to shore along a 2–3 m transect. Clean planktonic samples were filtered using a 100 μm hand net. Plankton samples containing debris and all benthic samples were refrigerated in their own water. Morphospecies were sorted as per the above protocols. At least 10 individuals per gross morphospecies were selected for preservation. Preserved specimens were stored at room temperature until August 3^rd^ 2010, at which point they were moved to −20°C freezer storage. Under a dissecting microscope (with 10-80X magnification range), two to three individuals per morphospecies per site per zone (planktonic/benthic) were selected for barcoding, for a total of 283 specimens. This field method considered which methods were appropriate for a given habitat, in that two methods were not employed for small and shallow habitats, and the project was focused on microcrustaceans.

### DNA barcoding

Specimen locality data, digital photos, and sequences were uploaded to the Barcode of Life Data Systems (BOLD) database [43]. All data are available as one dataset, entitled DS-Freshwater Ostracoda of Churchill [OSTCHU], accessible via the following permanent DOI (Will be assigned upon acceptance of the manuscript). The five different codes for the “Process IDs” (specimen identifiers assigned by BOLD to refer to the sequences for all specimens) reflect a total of 5 former projects on BOLD, each linked to one of the five collecting/sorting strategies (Table 1). DNA barcoding and sequence alignment was conducted according to standard methods [47], with the specifics of the protocols further described in Additional file 1: Appendix A.

For inclusion in analysis, sequences must have had >200 bp and <2% Ns. This is below the cut-off for the barcode standard for building a reference database of DNA barcodes (>500 bp and <1% Ns). However, shorter sequences can still be reliably matched to conspecifics [48]. All analyses were based on a provisional genetic definition of species, MOTUs. We named our MOTUs using sequential numbers added onto the institutional code for the Biodiversity Institute of Ontario, University of Guelph (e.g. Podocopid BIOUG001, etc.). MOTUs were assigned using Barcode Index Numbers (BINs) from BOLD3 [43], accessed March 16, 2012. BINs are genetic groupings assigned by BOLD for sequences >500 bp based on a 2% initial sequence divergence that is combined with an algorithm permitting deviations from this threshold in cases of genetic distance continuity or discontinuity [43]. We assigned our shorter sequences to these MOTUs if they clustered within a particular BIN on a neighbour-joining tree. The maximum pairwise sequence divergence within each MOTU, as well as the distance to the nearest neighbouring sequence belonging to a different MOTU, was calculated for all sequences of at least 500 bp using the Barcode Gap Analysis function in BOLD3 using the Kimura-2-parameter (K2P) distance model [49]. K2P distances have been more prevalent in the barcoding literature and were selected to facilitate comparison across studies. While two recent papers have argued for the use of p-distances instead, results using p-distances vs. K2P are very similar [50,51].

A neighbour-joining (NJ) tree was constructed in MEGA v. 5.0 using the K2P model for nucleotide substitutions and pairwise deletions of missing sites. One thousand bootstrap replicates were performed to assess nodal support of the clusters/MOTUs, without the assumption that this phenogram will accurately represent deeper phylogenetic relationships. Of 498 sequences recovered, 496 were used in the construction of this tree; 2 sequences were removed as they contained no overlapping sites with a number of other sequences and therefore would not allow distances to be calculated using MEGA. Clusters at the tips of the tree were collapsed for tree visualization.

### Statistical analyses

We performed basic summary statistics to elucidate the sequencing success rate for the specimens submitted for genetic analyses, as well as determined the number of genetic clusters recovered by each sampling strategy. To accommodate differing sample sizes, we compared provisional species richness among strategies using accumulation curves for successfully barcoded individuals. Curves were built in R version 2.14 [52] using the packages “picante” and “vegan” [53], with the curves randomized on a per-individual basis, without replacement, and with 1000 permutations. We further built curves that were rarefied by the number of sites sampled to ensure that there was no bias among studies having different numbers of sampling sites. Unless otherwise specified, sites without ostracods were excluded from their respective studies for analysis purposes. Curves based upon all sites would not be comparable because some studies were designed to be ostracod or microcrustacean focused, and therefore all or nearly all selected sites contained ostracods for those strategies. For the rarefied curves, we used the *specaccum* function in the package “picante”. We calculated Bray dissimilarity coefficients between the sampling strategies using normalized presence-absence data using the function *decostand* in the “vegan” package [53]. We performed 1-way ANOVAs and *ad hoc* tests of significance using the Bonferroni control to look for significant differences in dissimilarity between sampling strategies in their resulting species composition, i.e. genetic cluster composition. We did not perform an ANOVA on count data, but on dissimilarity coefficients. Upon plotting histograms of the dissimilarity values, they were approximately normally distributed.

### Species richness estimators

Even if species accumulation curves do not reach an asymptote, species richness estimators can be used to compare the richness of collections and to assess the stability of the biodiversity estimates with increasing sampling. We used the program EstimateS® version 8.2 [54] to compute the mean of the incidence-based, non-parametric richness estimators Chao2, first-order Jackknife (Jack1), and Incidence Coverage Estimate (ICE) for each sampling strategy, as well as the standard deviation for each and 95% confidence interval for Chao2. Chao2 is expected to be a robust and conservative estimate of diversity and uses the number of singletons and doubletons (species present in one or two samples) to infer the richness of additional species present but not detected [55]. We used the resulting indicators to generate plots showing total number of expected species with number of sites sampled, and compared the stability across the five strategies for the different richness estimators. We generated the set of richness estimators twice for the fixed-protocol and flexible-protocol strategies, once using only the total number of sites where ostracods were actually located and once using the total number of sites surveyed (refer back to Table 1).

## Results

We produced DNA barcodes for 498 individual Ostracoda, from an initial sample pool of 752 individuals selected for molecular analysis. This represented a sequencing success rate of 66.2%, which varied between 54% and 87% among the five strategies (Table 2). While we employed a cut-off of 200 base pairs for inclusion in this analysis, most sequences (97.4%) were >400 bp. We obtained 13 additional sequences >400 bp, but these were removed due to the presence of >2% ambiguous nucleotides (Ns).

**Table 2:** Summary of the primers used in each project and sequencing success rates

**Project**	**Primary primers**	**Secondary primers**	**Number of specimens**	**Success rate (%)**	**Number of specimens >200 bp with <2% Ns**
CHUBL	LCO1490_t1/HCO2198_t1	None	94	87%	78
SAOST	LCO1490_t1/HCO2198_t1	LCO1490_t1/MLepR1; MLepF1/HCO2198_t1^a^	95	83%	79
COCSA	LCO1490_t1/HCO2198_t1	CrustDF1/CrustDR1	190	65%	124
OZFWZ^b^	C_LepFolF/C_LepFolR	ZplankF1_t1/ZplankR1_t1	90	70%	63
OZFWC – Plates 1 and 2^b^	ZplankF1_t1/ZplankR1_t1	C_LepFolF/C_LepFolR	70	34%	24
OZFWC – Plates 3 and 4^b^	LCO1490_t1/HCO2198_t1	C_LepFolF/C_LepFolR	49	37%	18
OZFWC – Plates 5-9^b^	C_LepFolF/C_LepFolR	ZplankF1_t1/ZplankR1_t1	164	68%	112
**Combined**			**752**	**66.2%**	**498**

Primer sequences and references are provided in Additional file 1: Appendix A. The primer pairs used for both PCR amplification and cycle sequencing for each individual specimen are available through BOLD.

^a^ This primer combination involving mini-primers uses two separate PCR reactions to attempt to amplify the full-length barcode region in two fragments.

^b^ These samples were run on 96-well plates including mixed microcrustaceans, and thus the sample sizes do not reflect full plates.

**Figure 1:**
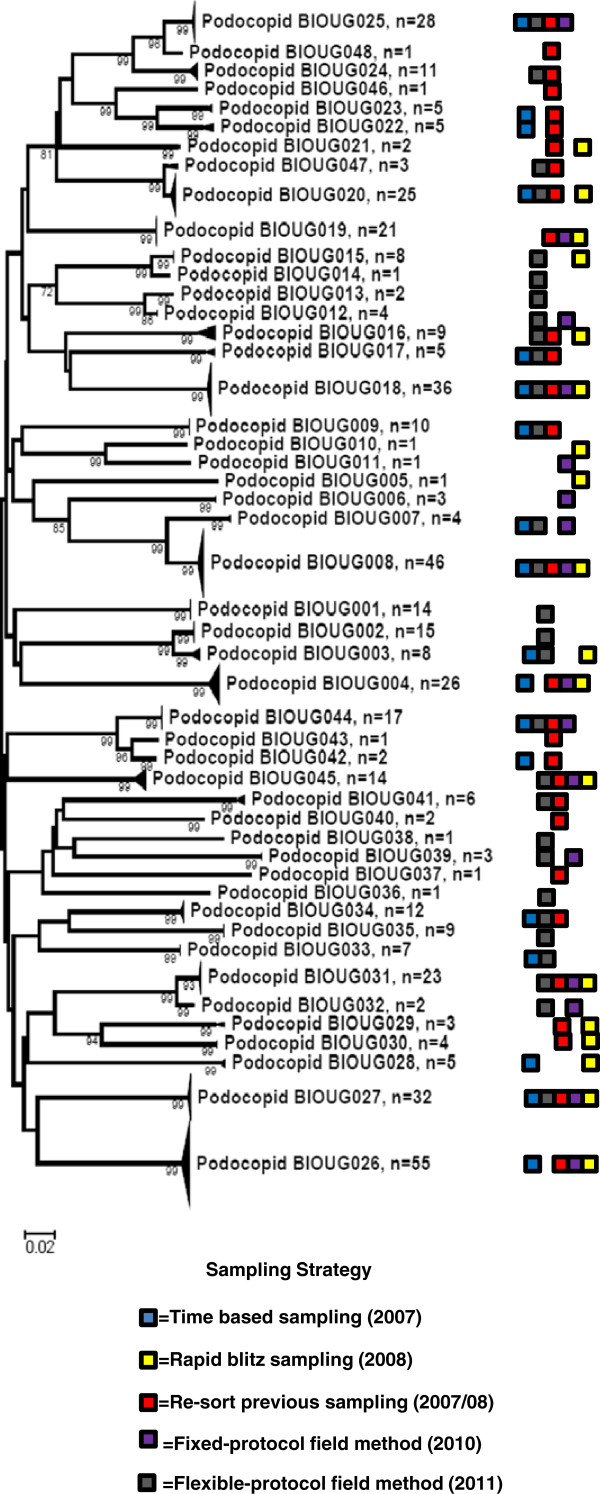
**Neighbour joining phenogram of ostracod specimens collected by sampling strategies denoting number of specimens per MOTU.** Each MOTU is labelled with coloured blocks indicating sampling strategies in which they were found.

We identified 48 genetic clusters of Ostracoda in the Churchill region from 2007 to 2011 (Figure 1), most of which were genetically differentiated from one another and well supported. The majority of the clusters that contained multiple individuals (35 of 38) were supported by high bootstrap values of >95%. Based upon sequences of at least 500 bp, maximum K2P distances within MOTUs ranged from 0–4.1%, with the mean of these maxima being 0.8%. All but four MOTUs had intra-MOTU maxima below 2%, and only 1 was above 2.6%. By contrast, inter-MOTU distances to the nearest neighbouring sequence of a different species ranged from 0.7-24.6%, with the mean being 12.3%. All but one of the nearest neighbour distances were >2.5%, with the exception involving a single poorly resolved MOTU pair having <70% bootstrap support for each cluster (MOTUs 020 and 048; Figure 1). This pair had an average distance of 1.9% between the two clusters, although some pairwise divergences were <2%.

### Presence/absence data

The liberal re-sort and flexible-protocol strategy captured 29 (~60%) of these clusters, while the time-based, rapid-blitz, and fixed-protocol strategies captured 18 (~38%), 18 (~38%), and 17 (~35%) MOTUs, respectively. Of the 48 genetic clusters identified in this analysis, only three (~6%) were captured by all five sampling strategies. Sixteen (~33%) genetic clusters were captured by only one of the five individual sampling strategies, and only 29 (~60%) clusters were captured by any two of the five sampling strategies. The flexible-protocol and liberal re-sort strategies proved highly efficient at capturing ‘rare’ clusters, here defined as appearing in only one or two of the five sampling strategies, but were also equally successful as the other sampling strategies at capturing common clusters, here defined as clusters appearing in three to five sampling strategies (Table 1). Of the 31 clusters appearing in only one or two sampling strategies, 14 and 15 (~45 and 48%) were identified in the liberal re-sort and flexible-protocol strategies respectively, compared to five or six (~16 or 19%) for each of the other strategies. The liberal re-sort and flexible-protocol also captured 83% of abundant clusters, compared to the remaining strategies, which captured 66-72% of abundant clusters.

### Accumulation curves

**Figure 2:**
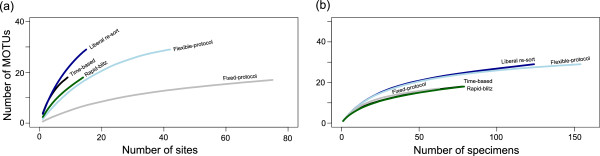
**MOTU accumulation curves for the different sampling strategies. a**. Site-based rarefaction curves for each sampling strategy for MOTUs. The total number of sites sampled is included for each strategy, including sites where no ostracods were found. **b**. Individual-based rarefaction curves for each of the sampling strategies for MOTUs. A curve is shown for each of the sampling strategies. Note that Figures 2a and 2b differ in scale for both the y and x axes.

The site-based rarefaction curves similarly revealed differences among the sampling methods and provided further differentiation among them (Figure 2a). At approximately 10 sites, the liberal re-sort emerged as the best method for maximizing MOTU richness, with the time-based method (focused on Ostracoda) being second. The flexible-protocol and rapid-blitz methods (both microcrustacean-focused) had intermediate effectiveness, with the fixed-protocol (and general invertebrate-focused) method being least effective. Moreover, this curve showed that the fixed-protocol strategy not only underestimated the number of genetic clusters found in this landscape but also gives the impression of approaching an asymptote, while the other methods continued to encounter additional species. When all sampling strategies are considered, it becomes apparent that there is a high level of diversity in this system captured by studies with fewer sampling sites than the fixed-protocol method, which appears to approach an asymptote due to methodological limitations. As studies differed in their target taxa and some included sites unsuitable for Ostracoda, we repeated this analysis including only sites containing Ostracoda. The fixed-protocol method remained the poorest in detecting ostracod MOTU richness, while the performance of the flexible-protocol method improved (results not shown). Individual-based rarefaction curves also showed differences in sampling effectiveness (Figure 2b). The liberal re-sort and flexible-protocol strategies emerged as superior for increasing MOTU richness with increasing sampling of individuals, while the other methods were similar. This means that MOTU richness for the liberal re-sort and flexible-protocol strategies increased with a greater slope with increasing sampling effort than the other strategies.

### Dissimilarity indices of methods

Bray dissimilarity coefficients were used to quantify the similarity between the sampling strategies (Table 3) in terms of MOTU composition. The strategies ranged from 36% similar to each other (rapid-blitz to flexible-protocol based) to 54% similar (time-based to intensive re-sorting). There were no significant differences in similarity comparisons between any of the strategies (mean P-value for ad hoc comparisons =0.055), with the majority of strategies being around 50% similar to each other. The time-based and rapid-blitz strategies were 54% and 50% similar, respectively, to the liberal re-sort method, which is made up of re-sorted specimens from those two strategies. When the time-based and rapid-blitz strategies were statistically pooled together, the resulting strategy remained 26% dissimilar from the liberal re-sort, showing that the liberal re-sort strategy captured additional richness initially missed by both strategies.

**Table 3:** Bray Curtis dissimilarities of distance comparisons of sampling methods

**Strategy**	**Compared with rapid blitz**	**Compared with liberal re-sort**	**Compared with fixed protocol**	**Compared with flexible protocol**
Time-based	0.55	0.46	0.55	0.52
Rapid-blitz	--	0.5	0.49	0.64
Liberal re-sort	0.5	--	0.61	0.51
Fixed-protocol	0.49	0.61	--	0.52
Flexible-protocol	0.52	0.51	0.51	--

Similarity between two sampling strategies is obtained from [1-Dissimilarity].

### Species richness estimators

**Figure 3:**
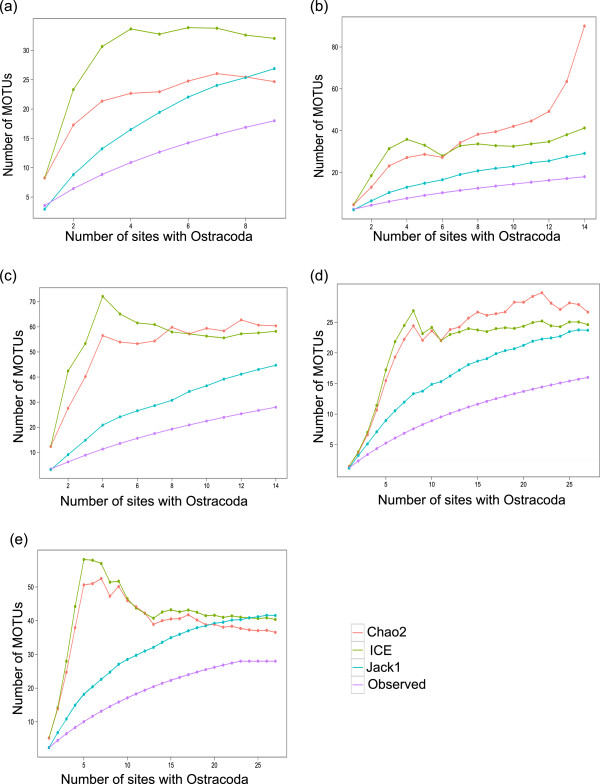
**MOTUs observed for the different strategies in comparison with richness estimates. a**) Time-based strategy; **b**) Rapid-blitz strategy; **c**) Liberal re-sort strategy; **d**) Fixed-protocol strategy; and **e**) Flexible-protocol strategy. Each point plotted represents the number of MOTUs generated in EstimateS for the corresponding number of sites containing Ostracoda.

There was variation among strategies in the MOTU richness estimators and their stability with increasing sampling (Table 4, Figure 3). For the time-based strategy (Figure 3a), the number of observed species does not approach the expected, as indicated by any of the richness estimators, by the total of nine sites sampled. Moreover, several of the estimators (Table 4) fall well short of the total observed richness for the Churchill region (total of 48 MOTUs for all 5 strategies). For the rapid-blitz strategy (Figure 3b), the Chao2 estimator of 90 is well beyond that of the observed total MOTU richness and does not appear to draw close to an asymptote. In the liberal re-sort, the observed richness falls short of the estimated (Figure 3c), as expected, but the estimators do reach asymptote by 14 sites sampled, with the Chao2 estimator of richness being 60.4 (reasonably above the observed total richness of 48). In comparing the fixed-protocol strategy (Figure 3d) and the flexible-protocol strategy (Figure 3e), both show stabilization of Chao2 and ICE, with the flexible-protocol method indicating lower richness estimates. Nine sites were not sufficient to estimate biodiversity in this system (thus excluding the time-based protocol). When all other strategies were compared at the 14 site mark, the rapid blitz (Figure 3b) and liberal re-sort (Figure 3c) provided the largest biodiversity estimators, with Chao2 of 90 and 60, respectively (Table 4). These are likely more accurate than the lower estimators, given that we have observed 48 MOTUs across strategies. Ten of these MOTUs are represented by a single specimen across all strategies.

**Table 4:** Mean MOTU richness estimates, standard deviation, and confidence intervals for each sampling strategy

**Strategy**	**Observed individuals (genetic)**	**Singletons mean**	**Doubletons mean**	**ICE mean**	**ICE SD**	**Chao2 mean**	**Chao2 SD**	**Chao2 95% CI**	**Jack1 mean**	**Jack1 SD**
Time-based*	18	10	5	32.0	0	24.7	5.7	19.6 – 46.6	26.9	4.5
Rapid-blitz*	18	12	1	41.3	0	90	83.5	29.8 – 458.2	29.1	4.3
Liberal re-sort*	29	18	5	58.2	4.9	60.4	1.8	37.8 – 135.4	44.7	7.4
Fixed-protocol▪	17	7.2	2.6	24.0	10.7	25.7	15.3	14.0 – 91.2	18.1	2.1
Flexible-protocol▪	29	13.3	5.5	42.6	15.1	40	13.9	26.4 – 90.4	33.6	3.8
Fixed-protocol^♦ ^	17	5.4	1.3	21.5	13.0	14.7	7.6	8.6 – 46.0	11.9	2.1
Flexible- protocol^♦ ^	29	12.8	3.5	56.6	47.5	41.0	16.2	24.6 – 97.6	29.1	4.3

To enable a comparison of strategies despite differences in number of sites sampled, we show here the diversity estimators for a mean of 14 sites sampled for all strategies except for time-based, for which the total number of sites sampled was nine. Fourteen was selected due to being the maximum number of sites available for two of the strategies, and nine sites (total for time-based) was recognized as being too few sites to accurately estimate richness for several of the strategies. By comparison, Chao2 and ICE stabilized at approximately 15 sites sampled for most strategies (Figure 3). Values are shown to one decimal place.

SD refers to standard deviation. CI refers to confidence interval.

*refers to strategies where the number of total sites sampled corresponds to the total number of sites where ostracods were found.

^■ ^ refers to results where only the total number of sites where ostracods were found was included.

^♦ ^ refers to results where the total number of sites sampled was included regardless of the presence of ostracods.

Richness estimators were also generated a second time for the fixed- and flexible-protocol methods using the total number of sites surveyed, regardless of whether they contained Ostracoda. Values for the richness estimators were similar in magnitude between the two datasets, but decreased for all metrics for the fixed-protocol strategy and increased for two metrics for the flexible-protocol strategy (Table 4).

## Discussion

### Comparisons of sampling strategies

This study presents an *a posteriori* assessment of the effectiveness of five freshwater ostracod collecting strategies, in order to help to inform the design of non-expert bioblitzes. We have found that the five sampling strategies differed both in the composition of the MOTUs they yielded and in total richness. We noted that only three MOTUs were found in all of the sampling strategies, and 16 MOTUs were only captured by a single strategy (Figure 1). Differences in composition were further verified by the Bray dissimilarity coefficients, which computed the dissimilarity of the different sampling strategies based on resulting MOTU composition. This analysis found low levels of similarity between the sampling methods (Table 3). This lack of similarity between sampling methods is not particularly surprising, however, considering that all strategies under sampled the total diversity. Moreover, we have confounded differences in site selection, some temporal differences between the different sampling strategies, as well as with some differences in equipment used. What is of greater interest in this study however, is the effectiveness of the different sampling strategies to capture a proportion of the total detected diversity and to serve as a predictor of total diversity in the region.

Both site-based and individual-based rarefaction curves revealed the liberal re-sort method to be superior in capturing total richness in this system. While it is not surprising that sequencing more specimens would yield more MOTUs, what was unexpected was that this pattern held *for a given sampling intensity*. This method was based upon field collections for two other strategies (time-based and rapid-blitz), which were either ostracod or microcrustacean focused or which aimed to include a variety of suitable microhabitats. This method involved more liberal inclusion of specimens for molecular processing and deliberately excluded one large-bodied, conspicuous (blue-coloured), and common morphospecies.

We also computed richness estimates using non-parametric methods for each of the sampling strategies. While these analyses showed that none of the employed sampling strategies captured the total richness present in the system, there was variability among the strategies in their ability to estimate richness. We conclude that the most appropriate of the five strategies for non-expert bioblitzes, in terms of both capturing and estimating diversity in this system, is the liberal re-sort because it captured the highest number of MOTUs, resulted in the second-highest Chao2 total richness estimator, and the Chao2 estimator remained stable after only 6 sites sampled. These results confirm that building upon field methods that included selecting a diversity of microhabitat and then more liberally including specimens for molecular analysis was most successful in characterizing diversity in this system.

### Implications of the evaluation of sampling strategies

In the past, deficiencies in freshwater ostracod sampling techniques led to the false conclusion that these animals were rare components of aquatic habitats [10]. While this is no longer the case, care must still be taken to use appropriate sampling strategies for ostracods. Two factors that influenced sampling effectiveness were whether morphospecies-based or liberal sorting of specimens was used, and whether microcrustaceans were the main goal of a collection. This study demonstrated that the greatest amount of ostracod diversity was uncovered in a microinvertebrate bioblitz aimed at the collection of specific taxa using an micro-habitat focused field strategy and a liberal sorting strategy, both of which allowed for higher numbers of specimens to be analyzed regardless of field morphospecies status. The two-phase method of a preliminary genetic analysis followed by a liberal resort (initiated specifically because of the detection of numerous singletons during phase 1) was an efficient way to direct resources. While we have provided partial evidence for this conclusion, we recognize that the differences among the five sampling strategies analyzed retrospectively in this paper (e.g., number of sites, season, equipment used, and PCR primers) may have affected our results. We suggest that partial evidence based upon considering available data can inform micro-invertebrate sampling design, pending further evidence from studies on other habitats and taxa. We recommend the following guidelines for designing non-expert biodiversity sampling programs that can use DNA barcoding for identification of samples: (a) Sampling strategies must include various macro and microhabitats. This study showed that non-experts were able to improve biodiversity estimators by actively selecting a variety of microhabitats. (b) A minimum of two sorting sessions should be done post collection when preparing specimens for DNA barcoding. The first session should include the dividing of organisms into gross morphospecies and selecting a representative number from each unit for DNA barcoding. A second, more liberal sorting strategy should include more replicates from the existing taxonomic units (as was the case in the intensive re-sort featured in this study); this sorting should be informed by phase one, as time and cost efficiency can be increased by deliberately excluding abundant morphospecies detected during phase one. (c) The objective of the study should be considered along with recognition of the benefits of a taxon-specific approach to collection compared to whole-community analyses. Whenever possible, sub-teams (even if all are non-experts) should focus on particular taxa within a broader whole-community analysis.

We note that our findings are applicable only to regions where the expected diversity of the system is relatively low (i.e. subarctic or temperate systems), thus facilitating reaching asymptotes in the biodiversity estimators, as we observed here. Understanding patterns of global diversity as well as alpha and beta diversity patterns would require far more sampling in tropical systems to elucidate regional biodiversity.

### Richness of the ostracoda of Churchill

This study also increased the known richness of the Churchill freshwater ostracod fauna. A previous multi-year study of ostracod diversity in the Churchill region by trained aquatic ecologists, including a multitude of sites and employing similar collection methods to our sampling strategies, yielded a total of 30 species identified through traditional taxonomy [1]. Through DNA barcoding, we have here identified 48 genetic clusters, which we interpret as provisional species due to comparison with genetic divergences in other microcrustaceans [e.g. [31]; this represents an increase in species richness of 56%. Nevertheless, the Churchill system is still undoubtedly under sampled for Ostracoda, as we found 10 MOTUs represented by a single individual across all strategies.

The prior underestimation of invertebrate species richness in this sub-arctic site is common to a variety of taxa. For example, the diversity of the crustacean class Branchiopoda has been characterized in Churchill using similar collecting methods as employed here, combined with DNA barcoding and morphological examination [31]. Interestingly, the known richness increased from 25 to 42 species/MOTUs [31], a similar proportional increase (68%) as we report here for the Ostracoda. This enhanced knowledge of MOTU richness in the microcrustaceans of Churchill, combined with their strong clustering patterns suggestive of species-level status of MOTUs, will be valuable for future studies of this site. Churchill has long been used as a model site for freshwater zooplankton community and population genetics studies [e.g. [1] and is now being developed as a site for comprehensive community studies through the creation of a comprehensive DNA barcode library [46].

### Next steps: whole-community bioblitzes?

DNA barcoding, arguably, originated with taxon-focused studies that consolidated data from many different sources [56]. Many of the early DNA barcoding papers were focused on defining methods and showing the efficacy of DNA barcoding for different taxonomic groups (e.g. [57]). In this way, early DNA barcoding was often focused on questions such as “how can we reliably differentiate closely related species?” This question and these seminal works were important for establishing DNA barcoding as a key technique in ecology and biodiversity studies, but the focus behind DNA barcoding has partially shifted in recent years. In addition to creating reference libraries and uncovering cryptic species or species complexes, DNA barcoding is increasingly being used to answer complex mechanistic ecological questions [e.g. [58-60]. The further shift from single-taxa to whole-community sampling [46] represents a significant trend in DNA barcoding research and one that is undoubtedly important. As DNA barcoding methods begin to be applied to whole communities to answer ecological questions, it is possible that despite increased taxonomic resolution with the genetic techniques, sampling programs aimed at whole communities may not be effective at capturing total diversity of certain groups. While collection efforts will remain a human effort, further advancement in next-generation sequencing technologies will contribute to the field of environmental DNA analysis [61], allowing for bulk sample analysis without the need for a sorting protocol. As demonstrated in this analysis, sorting protocol can have a profound impact on the results of a sampling strategy. After much-needed methodological advancements in understudied invertebrate taxa, environmental DNA analysis is poised to greatly contribute to community-level analyses of aquatic microinvertebrates [61,62].

While our study has shown definite differences in how much of the ostracod community each sampling strategy was able to capture, different strategies still have their merits for different study objectives and research programs. Storey et al. [63] tested two types of sampling methods for the collection of aquatic macroinvertebrates and noted that one method cost significantly less and was likely appropriate for research objectives such as long-term monitoring. The alternative method that they tested, however more costly and time consuming, proved to be more appropriate for studying new and unexplored areas [63]. This has implications for our earlier discussion of citizen science and the popularity of bioblitzes; while time-based or rapid-blitz approaches may be attractive to this type of citizen scientist sampling campaign because they allow large groups of participants to be involved simultaneously, they may not be the most appropriate depending on the goals of the study.

## Conclusion

While the main objective of this study was not to produce rigid rules for ostracod sampling programs conducted by non-experts, we nonetheless think that our findings have a place in current biodiversity science and the design of biodiversity sampling programs. By considering our findings, we hope that freshwater ecologists, and particularly those who engage in large-scale citizen science, can develop a set of best practices for these types of surveys and also recognize the utility of incorporating DNA barcoding into these programs. We found that sampling strategy had a substantial impact on species richness estimates of biodiversity surveys for a freshwater microinvertebrate fauna. Accumulation curves may approach an asymptote indicating completeness of sampling, but this can be attributed to artifacts of a sampling strategy limited not in scope but in methodological considerations. In our study, sampling strategies that were flexible in nature yielded higher species richness estimates than non-flexible sampling strategies, though consideration should be given to the benefits of a fixed-collection protocol in terms of comparative ecological analyses. We found that increasing the number of specimens analyzed during biodiversity surveys through the implementation of a liberal sorting strategy that is not reliant on morphospecies exposed additional diversity that was not originally appreciated by collectors based strictly on external morphological characteristics. This points to why DNA barcoding represents an excellent avenue with which to conduct biodiversity surveys of small-bodied organisms. Finally, strategies with an ostracod-specific or microcrustacean-focused collection mandate were more efficient than whole-community analyses at elucidating ostracod diversity on a per-site/time invested basis. Even with advanced molecular techniques, there should continue to be a balance when designing sampling programs.

## Authors’ contributions

BJL, AKW, and SJA conceived and designed the study. BJL and OZ completed the molecular laboratory analysis. BJL, AKW, EEB, and NWJ analyzed the data and prepared the figures. All authors conducted a portion of the field work and specimen sorting, wrote sections of the draft manuscript, and approved the final manuscript.

## Supplementary Material

Additional file 1 **Appendix A.** DNA barcoding lab protocols. Appendix B: List of primers used in this study. Click here for file
