# Supplementary material for: Insights into biodiversity sampling strategies for freshwater microinvertebrate faunas through bioblitz campaigns and DNA barcoding
Source: BMC Ecol. 2013 Apr 4;13:13. doi: 10.1186/1472-6785-13-13 (PMC3651337; doi:10.1186/1472-6785-13-13)
Supplement: Additional file 1 — Appendix A. DNA barcoding lab protocols. Appendix B: List of primers used in this study. [file 1472-6785-13-13-S1.doc]

**Appendix A – DNA barcoding lab protocols**

Each preserved specimen was first photographed and placed into an individual well in a 96-well lysis plate containing 50 uL of invertebrate lysis buffer combined with Proteinase K (20 mg/L) and incubated at 56°C for 12-16 hours**.** DNA was extracted and purified from individual specimens using the Glass Fiber method. Polymerase chain reaction (PCR) was used to amplify a ~710 base pair (bp) fragment of the 5’ region of the COI gene using standard primer pairs (Table 2; Appendix A). If the first primer set failed to amplify or sequence the COI fragment, additional primer sets were used (Table 2). The primers used for PCR and sequencing (along with all sequences, trace files, and collection data) are included for each specimen in the projects COCSA, OZFWC and OZFWZ in the BOLD database.

PCR mixes had a total volume of 12.5 μl and contained 6.25 μl 10% trehalose, 2 μl ddH_2_O, 1.25 μl 10X Buffer for Platinum *Taq*, 0.625 μl of 50mM MgCl_2_, 0.125 μl of each primer, 0.0625 μl 10mM dNTPs, and 0.06 μl Platinum *Taq* polymerase. Each well contained 2 μl of DNA template. The thermocycling profile consisted of the “COIfast” reaction, which cycled each plate for one cycle of 1 min at 94°C; 5 cycles of 94ºC for 1 min, 45ºC for 40 sec and 72º for 1 min; followed by 35 cycles of 94ºC for 1 min, 51ºC for 40 sec and 72ºC for 1 min; and then a final cycle of 72ºC for 5 min. When employing the zooplankton primers (ZplankF1_t1 and ZplankR1_t1), we used a modified thermocycling reaction which cycled each plate for one cycle of 1 min at 94°C; 40 cycles of 94°C for 40 sec, 52°C for 40 sec and 72°C for 1 min; and then a final cycle of 72°C for 5 min. PCR products were electrophoresed in a 2.0% agarose E-gel (Invitrogen) stained with ethidium bromide and were visualized under UV light.

Each PCR product was subjected to cycle sequencing and clean-up according to standard protocols employed at the Canadian Centre for DNA Barcoding and sequenced bi-directionally on an ABI 3730XL DNA Analyzer (Applied Biosystems). A single consensus sequence was assembled using the forward and reverse sequences using CodonCode Aligner v. 3.0.2 (CodonCode Corporation), and sequences were aligned using Clustal W in MEGA v. 5.0. Alignments were verified to be free of gaps and stop codons by using the amino acid translation.

Ivanova NV, DeWaard JR, Hebert PDN: **An inexpensive, automation friendly protocol for recovering high-quality DNA**. *Mol Ecol Notes* 2006, **6**:998-1002

**Appendix B**. List of primers used in this study.

| **Primer** | **Forward (F) or Reverse (R)** | **Sequence (5’-3’)** | **Note on usage** | **References** |
| --- | --- | --- | --- | --- |
| LCO1490_t1 | F | TGTAAAACGACGGCCAGTGGTCAACAAATCATAAAGATATTGG | PCR amplification; cycle sequencing reaction uses M13 tail sequence only | R. Floyd; Folmer et al. 1994; Messing 1983 (for M13 tail); |
| HCO2198_t1 | R | CAGGAAACAGCTATGACTAAACTTCAGGGTGACCAAAAAATCA | PCR amplification; cycle sequencing reaction uses M13 tail sequence only | R. Floyd; Folmer et al. 1994; Messing 1983 (for M13 tail); |
| C_LepFolF | F | LepF1: ATTCAACCAATCATAAAGATATTGG  LCO1490: GGTCAACAAATCATAAAGATATTGG | Cocktail of LepF1 and LCO1490 primers; for PCR and cycle sequencing | N. Ivanova (cocktail); Folmer et al. 1994; Hebert et al. 2004 |
| C_LepFolF | R | LepR1: TAAACTTCTGGATGTCCAAAAAATCA  HCO2198: TAAACTTCAGGGTGACCAAAAAATCA | Cocktail of LepR1 and HCO2198 primers; for PCR and cycle sequencing | N. Ivanova (cocktail); Folmer et al. 1994; Hebert et al. 2004 |
| ZplankF1_t1 | F | TGTAAAACGACGGCCAGTTCTASWAATCATAARGATATTGG | PCR amplification; cycle sequencing reaction uses M13 tail sequence only | Prosser, unpubl. |
| ZplanktR1_t1 | R | CAGGAAACAGCTATGACTTCAGGRTGRCCRAARAATCA | PCR amplification; cycle sequencing reaction uses M13 tail sequence only | Prosser, unpubl. |
| CrustDF1 | F | GGTCWACAAAYCATAAAGAYATTGG | PCR and cycle sequencing | Steinke unpubl. |
| CrustDR1 | R | TAAACYTCAGGRTGACCRAARAAYCA | PCR and cycle sequencing | Steinke unpubl. |
| MLepF1 | F | GCTTTCCCACGAATAAATAATA | PCR and cycle sequencing; most typically paired with a primer binding in the Folmer region | Hajibabaei et al. 2006 |
| MLepR1 | R | CCTGTTCCAGCTCCATTTTC | PCR and cycle sequencing; most typically paired with a primer binding in the Folmer region | Hajibabaei et al. 2006 |

Folmer O, Black M, Hoeh W, Lutz R, Vrijenhoek R: **DNA primers for amplification of mitochondrial cytochrome *c* oxidase subunit I from diverse metazoan invertebrates**. *Mol Mar Bio Biotechnol* 1994, **3:** 294-299.

Hajibabaei M, Janzen DH, Burns JM, Hallwachs W, Hebert PDN: **DNA barcodes distinguish species of tropical Lepidoptera**. *PNAS* 2006, **103**: 968-971.

Hebert PDN, Penton EH, Burns J, Janzen DH, Hallwachs W: **Ten species in one: DNA barcoding reveals cryptic species in the neotropical skipper butterfly, *Astraptes fulgerator***. *PNAS* 2004, **101**: 14812-14817.

Messing J: **New M13 vectors for cloning**. *Method Enzymol* 1983 **101**: 29–71.
